# Therapeutic target genes and regulatory networks of gallic acid in cervical cancer

**DOI:** 10.3389/fgene.2024.1508869

**Published:** 2025-01-20

**Authors:** Zhixi You, Ye Lei, Yongkang Yang, Zhihui Zhou, Xu Chao, Keyi Ju, Songyi Wang, Yuanyuan Li

**Affiliations:** ^1^ The Second Clinical Medical College, Shaanxi University of Chinese Medicine, Xianyang, Shaanxi, China; ^2^ The Second Affiliated Hospital, Shaanxi University of Chinese Medicine, Xianyang, Shaanxi, China

**Keywords:** gallic acid, RNA-sequencing, differentially expressed genes, candidate genes, regulatory network, cervical cancer

## Abstract

**Introduction:**

This study aims to identify the therapeutic targets and regulatory mechanisms of the antitumor drug gallic acid (GA) in cervical cancer (CC).

**Methods:**

HeLa cells were treated with GA and subjected to RNA-sequencing using the DNBSEQ platform. By combining the results of the Gene Expression Omnibus (GEO) and the Cancer Genome Atlas (TCGA) analysis and RNA-seq, the differentially expressed genes (DEGs), including those upregulated and downregulated genes in CC compared with the normal cervix in the GEO and TCGA database, while expressed reversed after treatment with GA, were identified. Subsequently, the function enrichment analysis and protein–protein interaction of the DEGs were conducted. The candidate genes were identified using the Cytoscape software Gentiscape2.2 and MCODE plug-ins. Furthermore, the upstream microRNA (miRNA), long noncoding RNA (lncRNA), and circular RNA (circRNA) of the candidate genes were predicted using the online tools of MirDIP, TarBase, and ENCORI. Finally, the regulatory network was constructed using Cytoscape software.

**Results:**

CC cells are significantly inhibited by GA. Combining the GEO and TCGA databases and RNA-seq analyses, 127 DEGs were obtained and subjected to functional enrichment analysis. This analysis revealed that 221 biological processes, 82 cellular components, 63 molecular functions, and 36 KEGG pathways were employed to identify three therapeutic candidate genes, including CDC20, DLGAP5, and KIF20A. The upstream 13 miRNAs, 4 lncRNA, and 42 circRNAs were detected and used to construct a lncRNA/circRNA-miRNA-mRNA-pathway regulatory network.

**Conclusion:**

This study identified candidate genes and the regulatory networks underlying the therapeutic effects of GA on CC using GA data mining methods, thus establishing a theoretical basis for targeted therapy of CC.

## 1 Introduction

According to the 2022 Global Cancer Statistics, cervical cancer (CC) is the fourth most common cancer and the fourth leading cause of cancer deaths in women worldwide (Bray et al., 2024). Globally, CC is the most common cancer of the female reproductive system and the second most common gynecological malignancy (Li et al., 2022; Zhao et al., 2023). Human papillomavirus (HPV) is a significant risk factor for CC. The development of an immune vaccine against HPV has reduced the incidence of HPV-associated CC cases by approximately 70% (Zhang and Batur, 2019). Despite the efficacy of these vaccines, CC remains a highly prevalent gynecological malignancy globally (Ahn et al., 2023; Wu L. et al., 2022). Furthermore, the vaccine does not prevent all subtypes of HPV infections. China has a low rate of HPV vaccination (Hu et al., 2021; Wu J. et al., 2022). Meanwhile, molecularly targeted therapies are becoming increasingly popular in tumor treatment (Vervoort et al., 2021; Zhang et al., 2022).

Gallic acid (GA), also known as 3,4,5-trihydroxybenzoic acid, exhibits antioxidant, anti-inflammatory, antidiabetic, antiangiogenic, antibacterial, antiviral, antifungal, anticarcinogenic, and other beneficial effects on human health (Hassani et al., 2023; Sagdicoglu Celep et al., 2022; Santana Andrade et al. 2022). GA can downregulate the molecular pathways involved in cancer progression, including the PI3K/Akt pathway (Ashrafizadeh et al., 2021). Furthermore, our findings indicate that GA can significantly inhibit the proliferation of several CC cell lines, including HeLa, SiHa, and C-33 A cells, in a dose-dependent manner.

This study aimed to identify the therapeutic molecular targets of GA in CC and the underlying regulatory mechanisms. To this end, HeLa cells were treated with GA and then subjected to RNA-sequencing (RNA-seq). Then, the RNA-seq results were combined with those obtained from the Gene Expression Omnibus (GEO) and Cancer Genome Atlas (TCGA) databases to identify differentially expressed genes (DEGs). Subsequently, the DEGs were analyzed for their functional enrichment and protein–protein interactions, resulting in the identification of therapeutic candidate genes and their upstream miRNA, lncRNA, and circRNA. Finally, a regulatory network was constructed. These findings provide a basis for further research into the regulatory mechanisms and molecular targets of GA in treating CC.

## 2 Materials and methods

### 2.1 Cell lines and cell culture

Three human CC cell lines, HeLa, SiHa, and C-33 A, were obtained from the American Type Culture Collection (ATCC, United States) and cultured in Dulbecco’s Modified Eagle Medium (DMEM) (Hyclone, United States) supplemented with 10% fetal bovine serum (FBS) (BI, United States) at 37°C with 5% CO_2_.

### 2.2 Cell viability assays

Cell viability was assessed using the Cell Counting Kit-8 (CCK-8, Sparkjade) assay. GA was dissolved in methanol at a concentration of 500 mM and then diluted to different concentrations (0 or control, 100 and 200 µM) using appropriate amounts of methanol. At the logarithmic growth stage, the cells were trypsinized, neutralized, and then seeded at a density of 5 × 10^3^ cells in 96-well plates containing complete DMEM. After 12–24 h, the culture medium was replaced with diluted GA. The absorbance of the plates was measured at 24, 48, and 72 h posttreatment at 450 nm. The data were analyzed using GraphPad Prism 9.0.0 software.

### 2.3 RNA Extraction, quantification, and sequencing

HeLa cells were cultured with the methanol control and a medium containing 200 μM GA (control and GA groups, respectively) for 3 days. When the cells reached a fusion of 60%–80%, they were collected and washed twice with PBS. Total RNA was extracted and purified from six samples (n = 3/group) using TRIzol Reagent (Invitrogen). RNA quantification and sequencing were performed using the BGISEQ-500 platform (BGI, China). The raw sequencing data were deposited in the Sequence Read Archive (SRA) of the National Center for Biotechnology Information (NCBI) under the accession number PRJNA1169526.

### 2.4 Microarray datasets

A total of four mRNA expression datasets obtained using human CC cell lines [GSE7803 (ZHAI et al., 2007), GSE9750 (Scotto et al., 2008), GSE63514 (den Boon et al., 2015), and GSE527 (WONG et al., 2003)] were selected and downloaded from the GEO database (https://www.ncbi.nlm.nih.gov/geo/). All datasets included analyses of the differences between normal cervical and CC tissue samples. GSE7803 is based on GPL96, [HG-U133A] Affymetrix Human Genome U133A array platform, which contains 10 normal squamous cervical epithelium and 21 invasive squamous CC samples. GPL96 was also used as the platform for GSE9750, which contains 24 normal cervical and 33 CC samples. The platform for GSE63514 is GPL570, [HG-U133_Plus_2] Affymetrix Human Genome U133 Plus 2.0 Array, which contains 24 normal cervical epithelium and 28 cervical squamous epithelial cancer specimens. GSE527 is the platform for GPL355, Human 10 K cDNA Array, which contains eight normal cervical and 25 CC samples. The RNA-seq results of HeLa cells treated with GA were provided by BGI.

### 2.5 Screening for DEGs

GEO2R (https://www.ncbi.nlm.nih.gov/geo/geo2r/) is an online analytical tool within the GEO dataset designed to analyze the DEGs between normal cervical and CC tissue samples. Genes were selected according to the following criteria: |logFC| ≥ 1, P-value < 0.05, and adj. P-value < 0.05.

The gene expression profiling interactive analysis (GEPIA2) (http://gepia2.cancer-pku.cn/#degenes) online database was used to analyze the DEGs in TCGA as follows: CESC, |Log2FC|Cutoff: 1, q-value Cutoff: 0.05, Differential Methods: ANOVA. The criteria for screening DEGs based on the RNA-seq results were as follows: DESeq2: |log2FC| ≥ 0.5, Q-value ≤ 0.05.

First, the significantly upregulated or downregulated genes among the four datasets of the GEO database were combined and duplicated, and the overlapping genes were removed. Then, a combined analysis of the results from the GEO and TCGA databases was used to screen common upregulated and downregulated genes between the CC and normal cervical samples. The duplicated genes were removed. Finally, by combining the results of the GEO and TCGA databases and RNA-seq, we identified genes that were upregulated or downregulated in CC compared with normal cervical samples, while the expression patterns of these genes were the reversed in GA-treated CC cells. The final differentially expressed genes are referred to as the “DEGs” in the following text for convenience.

### 2.6 Function analysis of the DEGs

The identified DEGs were subjected to Gene Ontology (GO, biological process, cellular component, and molecular function) and Kyoto Encyclopedia of Genes and Genomes (KEGG) pathway enrichment analysis using the Metascape database (https://metascape.org/gp/index.html). The enrichment criteria were as follows: Min overlap ≥3, P-value cutoff ≤0.05, min enrichment ≥1.5, and default values for the rest.

The Search Tool for the Retrieval of Interacting Genes (STRING, https://cn.string-db.org/) was employed to analyze and visualize the interaction relationship between proteins. The final set of DEGs was uploaded to STRING using the following settings: Meaning of network edges: confidence, Minimum required interaction score: high confidence (0.700), Network display options: disable structure previews inside network bubbles, hide disconnected nodes in the network.

### 2.7 Screening for therapeutic candidate genes of GA

Gene sets with interaction relationships were imported into the Cytoscape software to create a visible network. First, the Gentiscape2.2 plug-in was employed to calculate the network and topological characteristics of each node. The genes corresponding to the nodes with degree value ≥Mean + SD and Betweenness value ≥Mean + SD nodes were referred to as hub and bottleneck genes, respectively. With the MCODE plug-in, the parameters were set as degree cutoff ≥3, K-core ≥4, and default values for the rest. The core genes that constitute the stable network structure were screened out. Finally, the potential therapeutic candidate genes that were in common among the hub, bottleneck, and core genes were identified using the “Calculate and draw custom Venn diagrams” online tool (http://bioinformatics.psb.ugent.be/webtools/Venn/).

### 2.8 Quantitative real-time PCR (qRT-PCR) analysis

The total RNA was reverse transcribed to cDNA using the RevertAid First Strand cDNA Synthesis Kit (Thermo Fisher Scientific), which was used as a template for qRT-PCR amplification. The PCR mixtures were prepared to a final volume of 20 µL using the PowerUpTM SYBRTM Green Master Mix (Applied Biosystems) with four wells for each sample. The PCR assay was performed using the ABI Q5 real-time PCR platform (ABI Life Technologies), and the cycling parameters were as follows: 1 cycle of 50°C for 2 min, 1 cycle of 95°C for 2 min, 40 cycles of 95°C for 15 s and 60°C for 1 min. Fold changes were calculated and normalized via the ΔΔCt method using the glyceraldehyde 3-phosphate dehydrogenase gene as the internal normalization control. The primers used were as follows: (5ʹ-3ʹ): CDC20 (F: CTG​GAT​CAA​AGA​GGG​CAA​CTA, R: GGC​AGA​GTG​ACT​GGT​CAT​ATT); DLGAP5 (F: GTT​GTG​CAG​CCT​GTA​ATG​CC, R: TAG​CAG​CTC​TTG​TGA​CTG​GC); KIF20A (F: TGC​TGT​CCG​ATG​ACG​ATG​TC, R: AGG​TTC​TTG​CGT​ACC​ACA​GAC); and GAPDH (F: CAA​TGA​CCC​CTT​CAT​TGA​CC, R: GAC​AAG​CTT​CCC​GTT​CTC​AG).

### 2.9 Upstream miRNAs, lncRNAs, and circRNAs analysis of candidate genes

The candidate genes were submitted to the MirDIP (http://ophid.utoronto.ca/mirDIP/index_confirm.jsp), miRDB (https://mirdb.org/), and ENCORI (https://rnasysu.com/encori/) online databases to predict their upstream interaction with miRNAs. Then, the reliability of the miRNAs was verified using TarBase V 9.0 software (https://dianalab.e-ce.uth.gr/tarbasev9). The miRNAs were submitted to the ENCORI online tool for analyzing the relationships between miRNA and lncRNA (parameters setting: Clip-data ≥2 datasets, Degradome Data ≥1), and miRNA and circRNA (parameter setting: Clip-data ≥2 datasets, Degradome Data ≥3, and default value for the rest).

### 2.10 Construction of the LncRNA/CircRNA-miRNA-mRNA-pathway regulatory network

The relationships between the upstream lncRNAs and circRNAs with miRNAs, miRNAs with candidate genes, and the candidate genes with pathways that met the screening criteria were evaluated. The file was imported into the Cytoscape software to establish a visual lncRNA/circRNA-miRNA-mRNA-pathway regulatory network.

### 2.11 Statistical analysis

The data were analyzed using GraphPad Prism 9.0.0 software (La Jolla, CA, United States) (https://www.graphpad.com). The control and GA groups were compared using the Student’s t-test. A significant difference (P < 0.05) was indicated between the groups based on the means +SEM.

## 3 Results

### 3.1 GA inhibited the proliferation of CC cells

**FIGURE 1:**
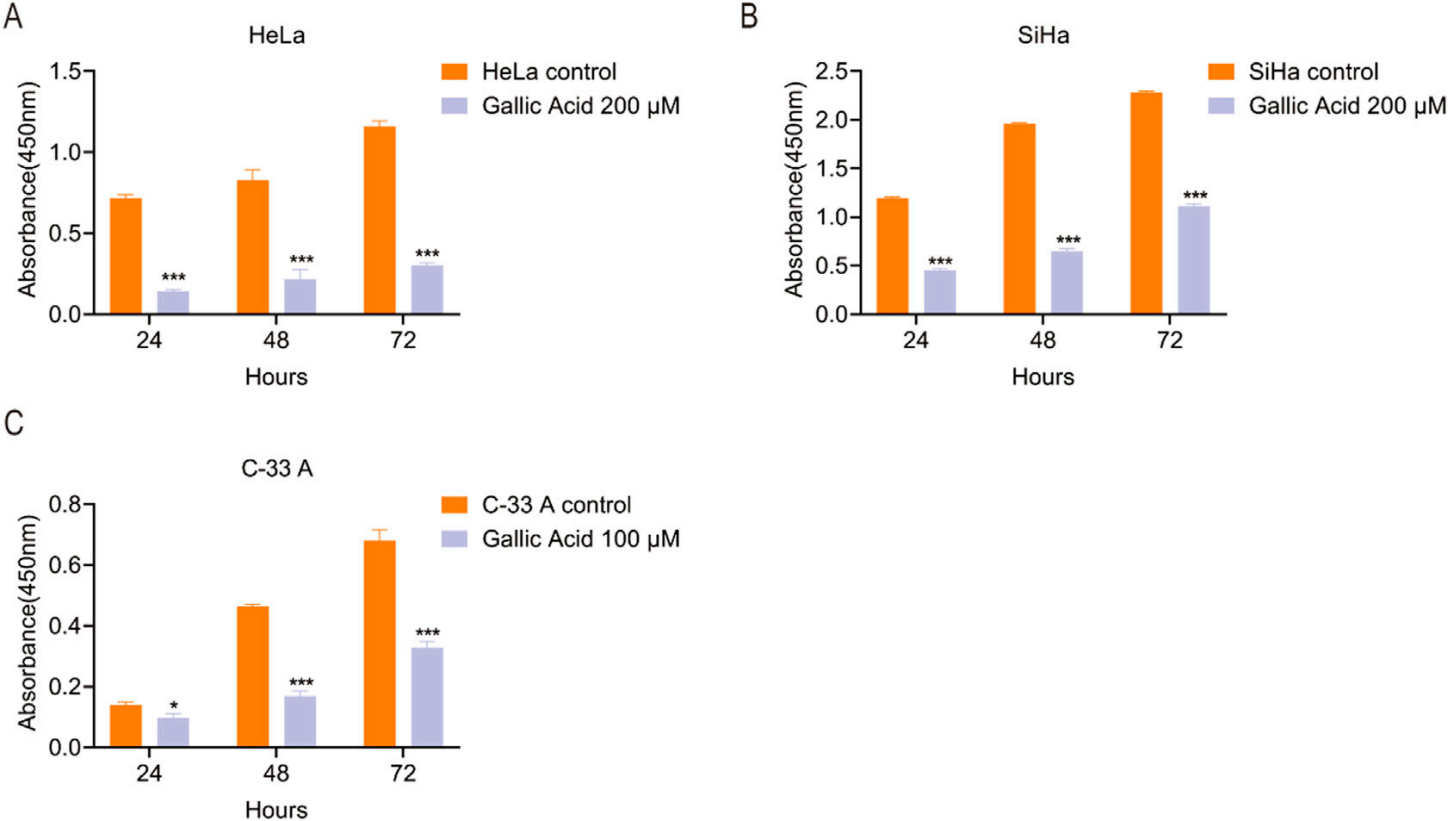
Gallic acid inhibits the proliferation of cervical carcinoma cells. **(A)** HeLa, **(B)** SiHa, and **(C)** C-33 A cells were treated with methanol vehicle control and diluted gallic acid, and their proliferation was assessed using the CCK-8 assay. The values are shown as the mean ± SEM (Student’s t-test was used for the statistical analysis (*p < 0.05, **p < 0.01, ***p < 0.001 vs. the corresponding control).

The CCK-8 assay was used to assess the proliferation of HeLa, SiHa, and C-33 A cells treated with different GA concentrations (0, 100, or 200 µM) was assessed. The results indicated that GA significantly inhibited the proliferation of CC cells (Figure 1).

### 3.2 Identification of DEGs in the treatment of CC with GA

**FIGURE 2:**
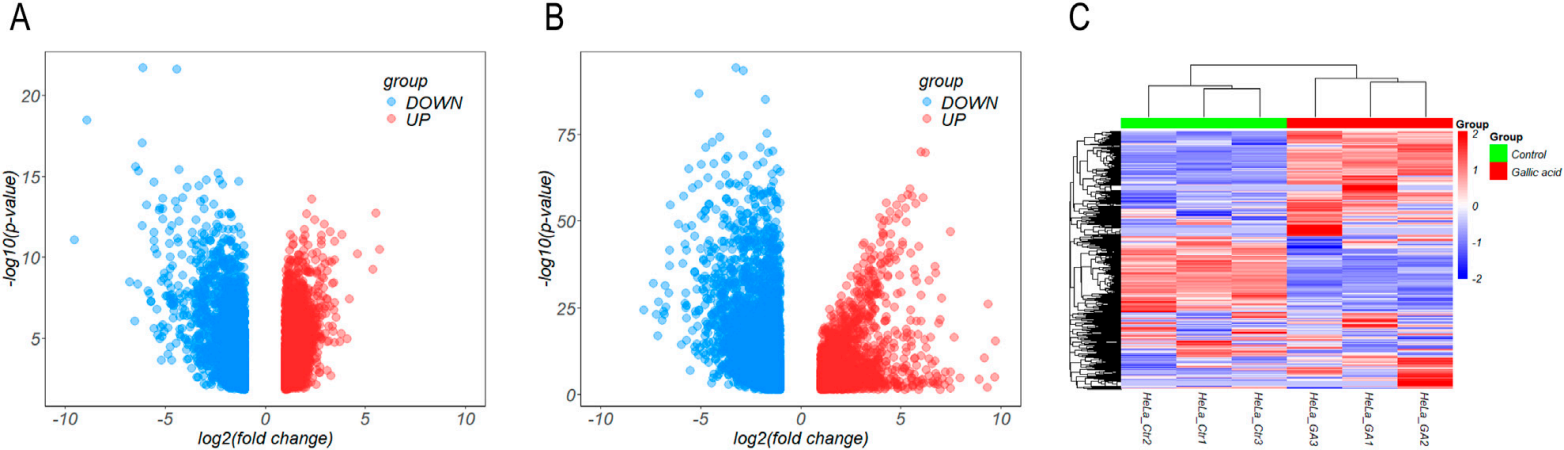
Analysis of differentially expressed genes. **(A, B)** A volcano plot of differentially expressed genes in Gene expression omnibus **(A)** and the Cancer Genome Atlas **(B)** databases. **(C)** Heat map comparing the control and experimental groups of cervical cancer cells treated with gallic acid.

Four raw expression microarray datasets of CC (GSE7803, GSE9750, GSE63514, and GSE527) were analyzed using GEO online. After filtering out 64 genes with opposite expression trends, we screened 5,234 DEGs between normal cervical and CC tissue samples, including 2,806 upregulated and 2,428 downregulated genes (Figure 2A).

In the TCGA database, 6,072 genes were screened out (three duplicates and one gene with opposite expression removed), including 2045 upregulated and 4,027 downregulated genes. Combining the results of both databases, we screened the genes with the common expression trend, which included 629 upregulated and 630 downregulated genes (Figure 2B).

As GA significantly inhibited the proliferation of CC cells, we treated HeLa cells with GA and then performed an RNA-seq analysis to identify the target genes. A total of 2,343 DEGs were screened out, including 1,501 upregulated and 842 downregulated genes (Figure 2C).

Combining the results from the GEO and TCGA databases with RNA-seq, we identified the upregulated and downregulated genes in CC compared with normal cervical samples. After treatment with GA, the expression of these genes was reversed, with downregulated genes becoming upregulated and *vice versa*. Finally, we screened out 127 DEGs, including 40 upregulated and 87 downregulated genes, in the GEO and TCGA databases, which were downregulated and upregulated after treatment with GA, respectively, showing an opposite trend (Table 1).

**TABLE 1:** Screening 127 differentially expressed genes (DEG) combining the GEO, TCGA database and RNA-Seq results.

A. 40 genes upregulated in GEO and TCGA, while downregulated after treated with gallic acid
ASPM, BRIX1, BUB1, CCNE2, CCNF, CDC20, CEBPG, CENPE, DEPDC1, DEPDC1B, DKC1, DLGAP5, EIF4EBP1, FAM111B, FAM72C, GINS2, HMGB3, KIF20A, KRT17, LMNB1, LRRC8B, LYN, MANEAL, MCM6, MET, MKI67, MTHFD1L, NLN, PIF1, PSAT1, RAB3IP, SHMT2, SLC16A3, SLC38A1, SYNGR3, TEAD4, TIPIN, TMPO, TYMP, UBE2S

B. 87 genes downregulated in GEO and TCGA, while upregulated after treated with gallic acid
ACSM3, ACVRL1, AGFG2, AOX1, AQP1, ARHGAP6, ARPC4-TTLL3, ATOH8, C14orf132, CFAP70, COL14A1, COL21A1, COL6A1, CPA3, CRMP1, CYP11A1, DCLK1, DCN, DKK3, ECM2, EFEMP2, FAM153A, FAM214A, FAXDC2, GNG11, GRIK5, GRIP2, GSN, HIC1, HPGD, HPSE2, IGF2, IGFBP6, IL1R1, KALRN, KCNAB1, KCTD17, KLF8, LAMA2, LRRK2, MAOB, MASP1, MATN2, MEOX1, METTL7A, MFNG, MRGPRF, MROH7, MSX1, MXD4, MYZAP, NACAD, NFASC, NFATC4, PDK4, PHYHIP, PKD1L2, PLCD1, PLXNA4, PPP1R3C, PROS1, PRPH, PTGDS, PTN, QPRT, RGS11, RHBDF1, SBSPON, SH3BGRL2, SHC3, SLC11A1, SLC27A1, SLC7A8, SLCO2A1, SPON1, SYNGR1, TACR1, TCP11L2, THSD4, TIMP4, TNXB, TOM1L2, TPRG1, TRIOBP, TTLL3, TUBA1A, VAT1

### 3.3 GO function and KEGG pathway enrichment analysis of the DEGs

The DEGs were uploaded to the Metascape database for GO (biological processes, cellular components, and molecular functions) and KEGG pathway enrichment analysis.

**FIGURE 3:**
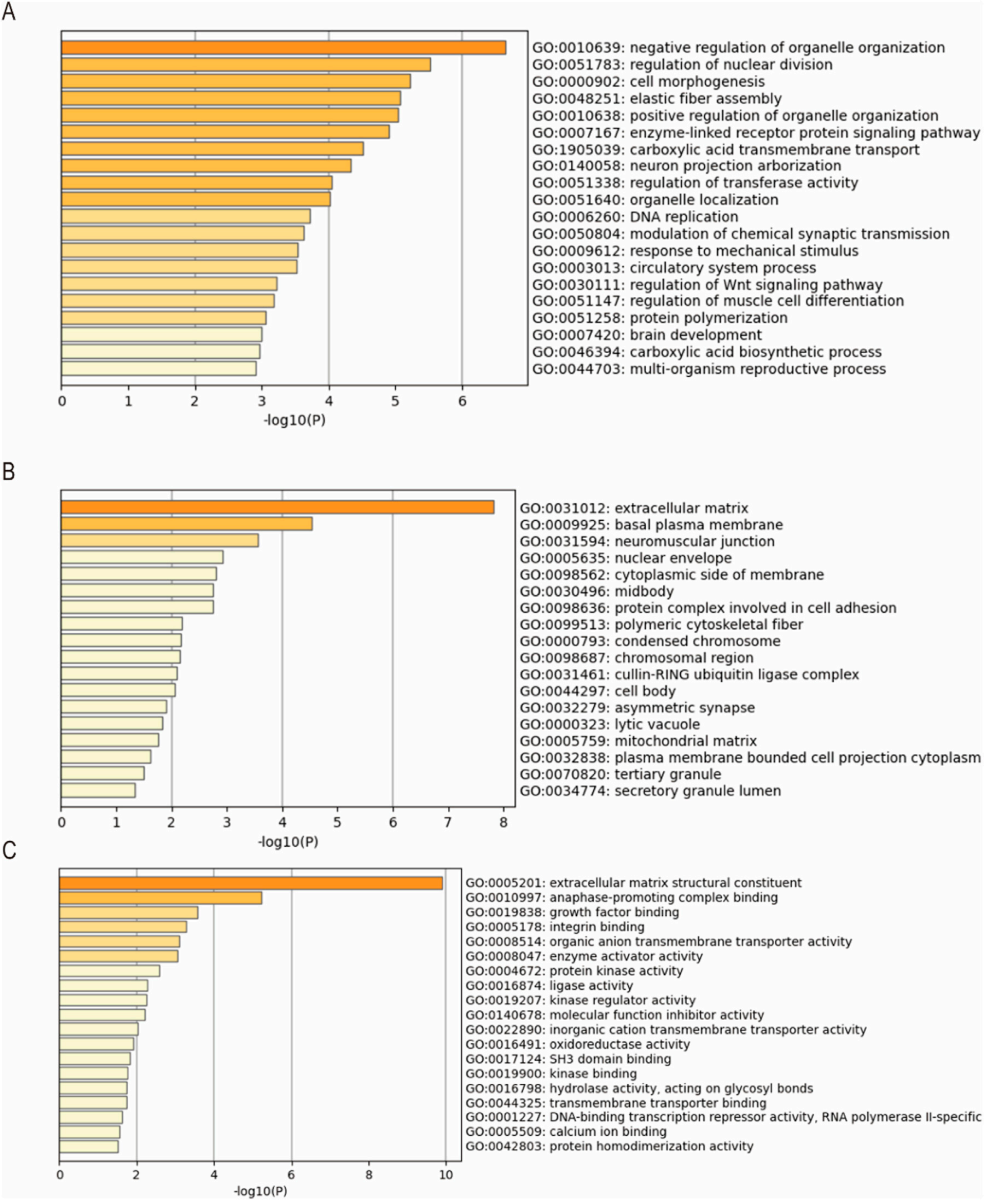
Gene Ontology (GO) enrichment analysis results of the DEGs. The top **(A)** 20 clusters of biological processes, **(B)** 18 clusters of cellular components, and **(C)** 19 clusters of molecular functions with their representative enriched terms.

A total of 221 biological processes, 82 cellular components, and 63 molecular functions were enriched. The top 20 clusters of biological processes are shown in Figure 3A, such as negative regulation of organelle organization, regulation of nuclear division, cell morphogenesis, elastic fiber assembly, and positive regulation of organelle organization. The top 18 clusters of cellular components, as shown in Figure 3B, included the extracellular matrix, basal plasma membrane, neuromuscular junction, nuclear envelope, and cytoplasmic side of the membrane. As shown in Figure 3C, the top 19 clusters of molecular function included extracellular matrix structural constituents, anaphase-promoting complex binding, growth factor binding, integrin binding, and organic anion transmembrane transporter activity.

**FIGURE 4:**
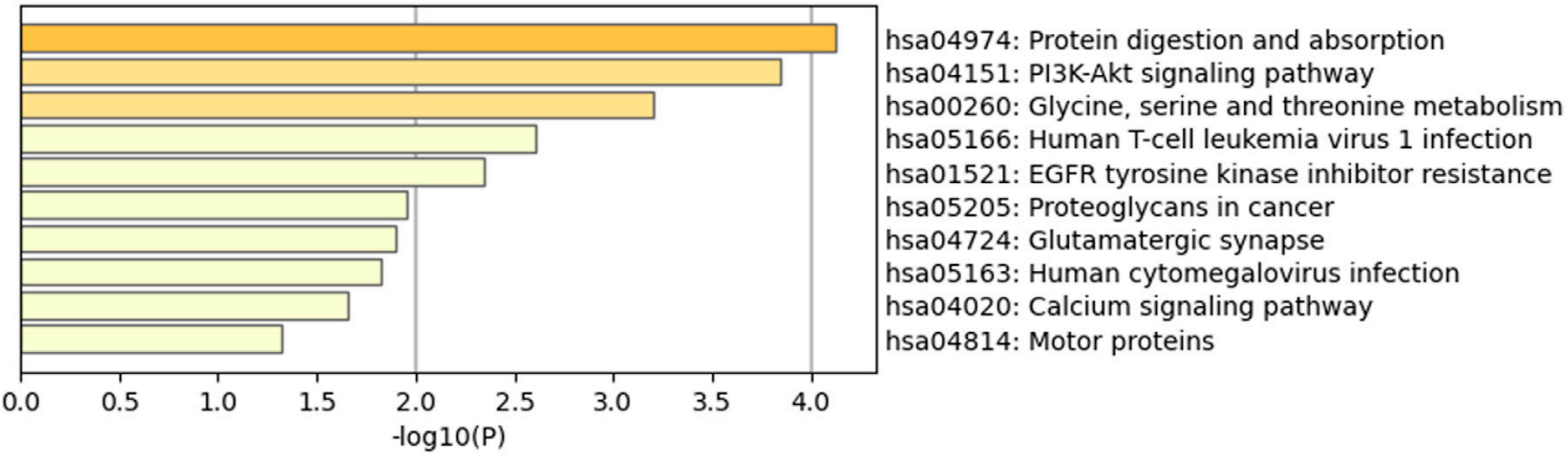
Kyoto Encyclopedia of Genes and Genomes (KEGG) pathway enrichment analysis results of DEGs. Of the 36 KEGG pathways enriched, the top 10 clusters are shown with their representative enriched terms.

Furthermore, 36 KEGG pathways were enriched, and the top 10 clusters with their representative enriched terms included protein digestion and absorption, the Pl3K-Akt signaling pathway, “Glycine, serine and threonine metabolism,” human T-cell leukemia virus 1 infection, and EGFR tyrosine kinase inhibitor resistance (Figure 4).

**FIGURE 5:**
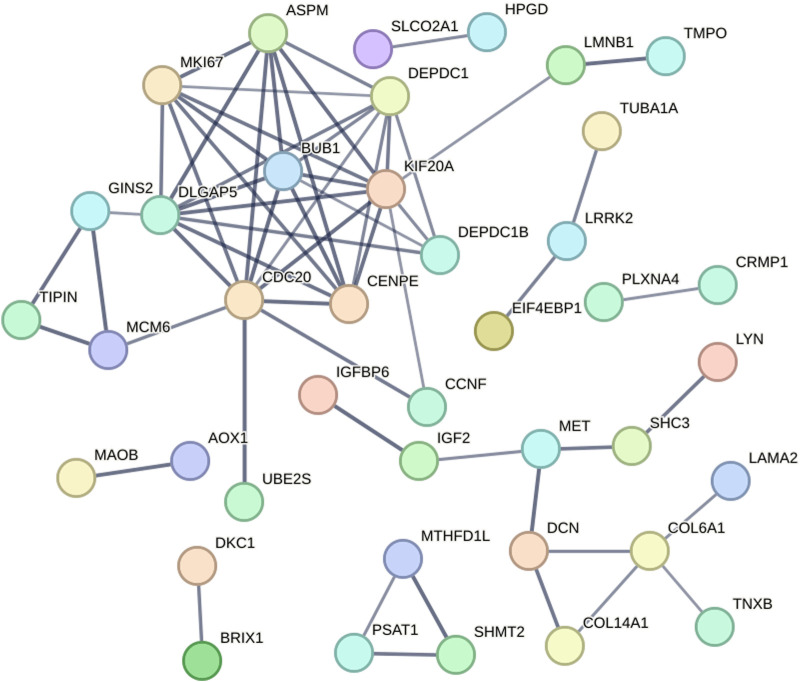
Protein–protein interaction analysis of the DEGs. A protein interaction network with 61 edges of the remaining 40 node-proteins was constructed.

The protein–protein interaction relationships corresponding to the DEGs were analyzed and visualized using the STRING online tool. According to the setting parameter criteria, 87 proteins without interaction were hidden, and a protein interaction network with 61 edges of the remaining 40 node-proteins was constructed (Figure 5).

### 3.4 Screening of therapeutic candidate genes and analysis of upstream MiRNA, LncRNA, and CircRNA

**FIGURE 6:**
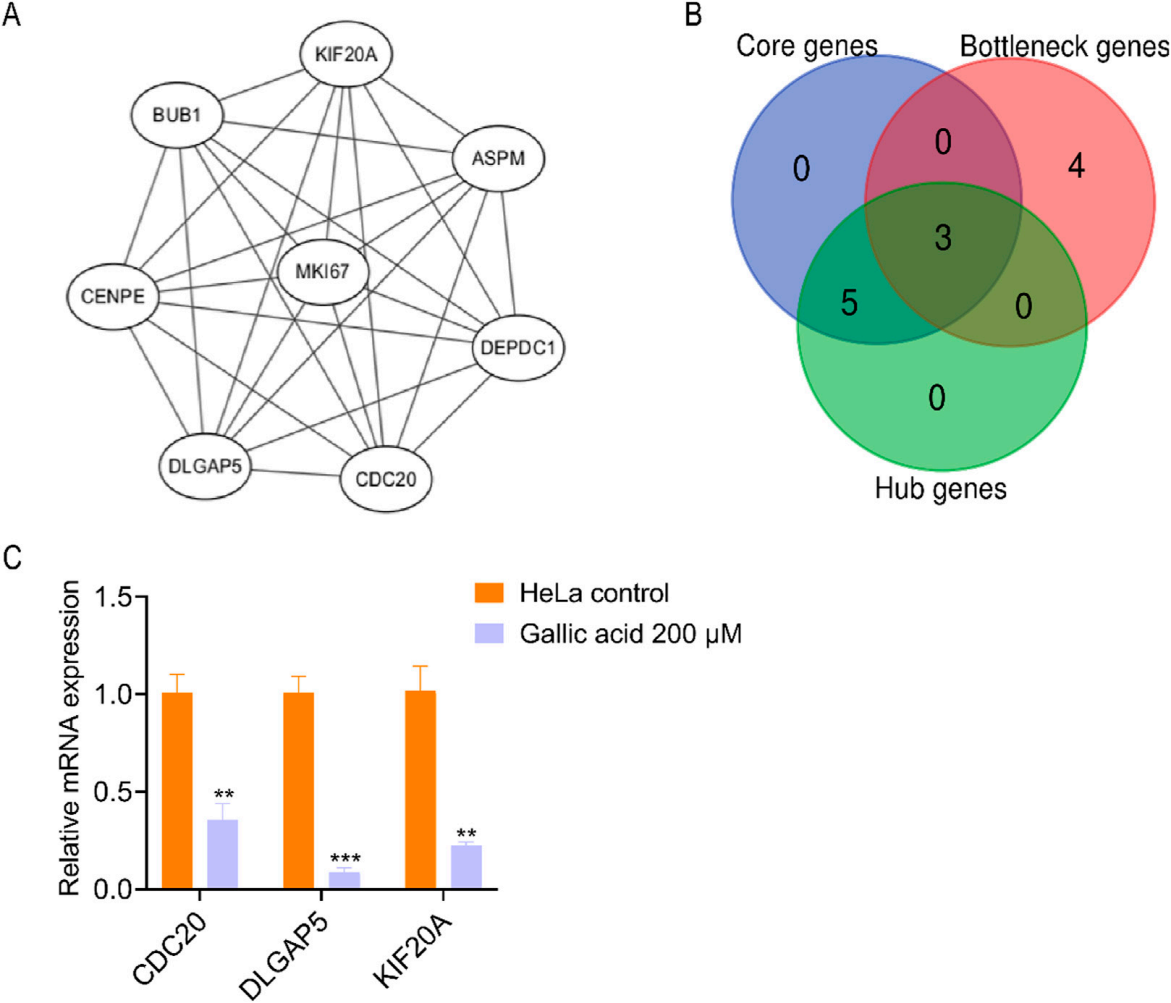
Screening therapeutic candidate genes. **(A)** Interaction diagram of the core genes. **(B)** Venn analysis of the core genes, hub genes, and bottleneck genes. **(C)** The mRNA expression levels of the common genes analyzed using qRT-PCR assay after treating HeLa cells with methanol control and 200 μM gallic acid. The statistical analysis was shown. Student’s t-test was used for the statistical analysis, **p < 0.01, ***p < 0.001.

The protein–protein interaction network was imported into the Cytoscape software with the MCODE plug-in. The eight core genes constituting the stable structure of the network were screened out (Figure 6A; Table 2). Using the Gentiscape2.2 plug-in, eight hub and seven bottleneck genes were screened out (Table 2). The core, hub, and bottleneck genes, which were in common and are critical to the network, were considered the candidate genes. These included cell division cycle 20 (CDC20), disks large homolog associated protein 5 (DLGAP5), and kinesin family member 20A (KIF20A) (Figure 6B; Table 2). We used real-time PCR to detect the RNA expression levels of the candidate genes in the HeLa control and GA groups. The results showed that the expression of three candidate genes (CDC20, DLGAP5, KIF20A) matched the RNA-seq analysis (Figure 6C).

**TABLE 2:** Screening result of therapeutic candidate genes.

Types	Names
Core genes	ASPM, BUB1, CDC20, CENPE, DEPDC1, DLGAP5, KIF20A, MKI67
Hub genes	CDC20, KIF20A, DLGAP5, BUB1, DEPDC1, ASPM, CENPE, MKI67
Bottleneck genes	CDC20, KIF20A, MET, DCN, DLGAP5, COL6A1, LMNB1
Candidate genes	CDC20, DLGAP5, KIF20A

Furthermore, the three candidate genes were submitted to the MirDIP, miRDB, and ENCORI online databases to predict their upstream miRNAs. Reliability was verified using TarBase V9.0 software, and 13 miRNAs were screened (Table 3). Subsequently, these miRNAs were submitted to the ENCORI database and four upstream lncRNAs and 42 circRNAs were obtained after removing duplicate genes.

**TABLE 3:** Analysis of miRNA, lncRNA, circRNA of upstream interaction of candidate genes.

Types	Names
miRNA	hsa-miR-147a, hsa-miR-210–3p, hsa-miR-25–3p, hsa-miR-29a-3p, hsa-miR-29c-3p, hsa-miR-32–5p, hsa-miR-34a-5p, hsa-miR-374a-5p, hsa-miR-374b-5p, hsa-miR-378a-5p, hsa-miR-629–5p, hsa-miR-92a-3p, hsa-miR-92b-3p
lncRNA	KCNQ1OT1, MAGI2-AS3, MALAT1, SNHG7
circRNA	HDGF, HDLBP, IARS, TPT1, DCXR, ELAVL1, NDUFS6, SH3GLB2, MGRN1, OTUD3, ATP5G3, C5orf24, ZDHHC5, RECQL5, ENO1, EDARADD, HNRNPH1, SUN1, SET, COL5A1, RPS24, KIAA1598, C11orf10, AHNAK, RPLP0, TPM1, TRIM28, MTSS1L, ATP5F1, LAMB2, ALG3, POM121, CASP2, EI24, GAPDH, P2RX5-TAX1BP3, SRSF7, ANXA1, HSBP1, LUZP6, MTPN, USP9X

### 3.5 lncRNA/CircRNA-miRNA-mRNA-pathway regulation network

**FIGURE 7:**
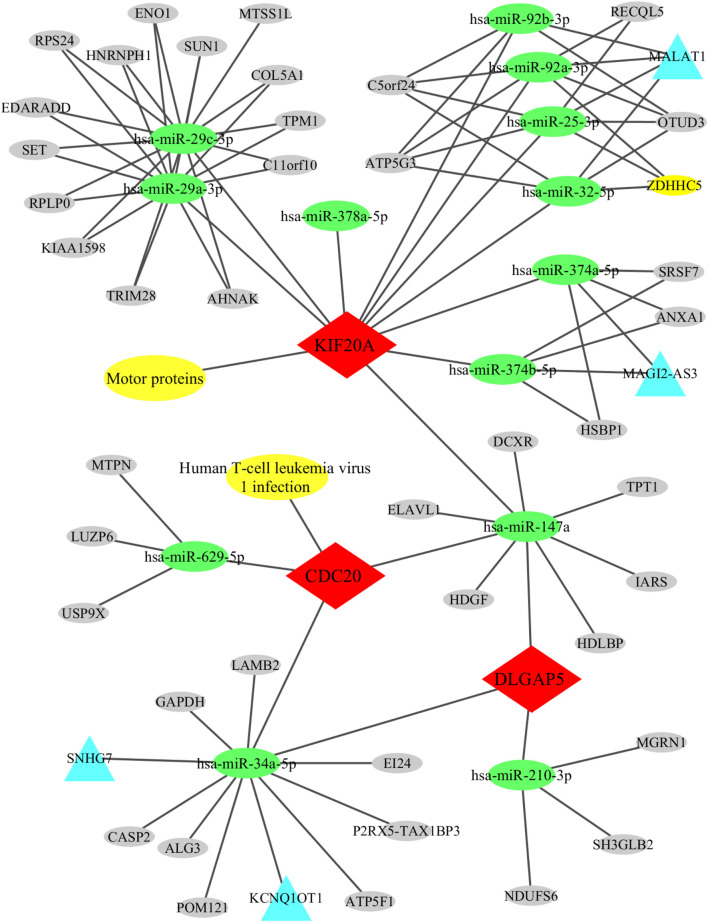
Regulatory network of lncRNA/circRNA-miRNA-mRNA. This pathway consists of 63 nodes (3 candidate genes, 1 KEGG pathway, 13 miRNAs, 4 lncRNA, 42 circRNAs) and 95 edges.

All relationships, including the candidate genes with their associated KEGG pathways, the upstream lncRNAs and circRNAs with the miRNAs, and the miRNAs with the candidate genes, were imported into the Cytoscape software for constructing and visualizing the lncRNA/circRNA-miRNA-mRNA-pathway regulatory network. This network comprised 63 nodes (3 candidate genes, 1 KEGG pathway, 13 miRNAs, 4 lncRNA, 42 circRNAs) and 95 edges (Figure 7).

## 4 Discussion

Multiomics and multilevel biomedical data enable a holistic understanding of different biological mechanisms. Biomedical data are widely dispersed and complex; thus, bioinformatics is increasingly being used to diagnose and treat various diseases.

TCGA and GEO are the two most widely used tumor databases. In the GEO database, four raw expression microarray datasets related to CC were examined (GSE527, GSE7803, GSE9750, and GSE63514), excluding 64 genes with conflicting expression patterns. In the TCGA online database, 6,077 genes, 3 duplicates, and one gene with opposite results were also removed.

Using the Cytoscape software Gentiscape2.2 and MCODE plug-ins, we screened three candidate genes (CDC20, DLGAP5, and KIF20A) involved in the therapeutic effects of GA on CC and predicted their upstream miRNAs using the MirDIP and TarBase online tools. CDC20 is highly expressed in several tumor types, making it a potential therapeutic target for cancer (Jeong et al., 2022; Tsang and Cheeseman, 2023; Zhang et al., 2019). In patients with mantle cell lymphoma, high CDC20 expression correlated with unfavorable clinicopathological features and poor prognosis (Chen et al., 2023). There is a significant correlation between the expression of DLGAP5, which is highly expressed in many cancer types, and poor prognoses in patients with cancer (Chang et al., 2022). Moreover, DLGAP5 promotes the proliferation and invasion of hepatocellular carcinoma (HCC) (Tang et al., 2021), bladder cancer (BLCA) (Zhou et al., 2024), and breast cancer (Li et al., 2023). KIF20A is highly expressed in cancer cells, making it a promising therapeutic target for various cancers (Jin et al., 2023). KIF20A promotes the progression of castration-resistant prostate cancer by activating androgen receptor signaling (Copello and Burnstein, 2022) and promotes the progression of fibrosarcoma via the PI3K-Akt signaling pathway (Jin et al., 2022). After analyzing the upstream lncRNAs and circRNAs of the miRNAs using the ENCORI online tool, we imported the identified relationships into Cytoscape to create a visual regulatory network involving lncRNAs, circRNAs, miRNAs, mRNAs, and the molecular pathways.

## 5 Conclusion

In this study, we demonstrated that GA inhibits the proliferation of CC cells via cell biology experiments. Furthermore, using bioinformatics methods and RNA-sequencing, we identified candidate genes and associated regulatory networks underlying the therapeutic effects of GA in CC. Real-time quantitative PCR results confirmed the significant differences in the expression of these candidate genes. However, further investigations are required to elucidate the mechanisms underlying the therapeutic effects of GA on CC. Additionally, to comprehensively evaluate the therapeutic efficacy of GA against CC, more animal models and clinical trials are essential to assess its safety, optimal dosage, administration routes, and treatment duration.

## Data Availability

The raw sequencing data generated in this study have been deposited in the NCBI Sequence Read Archive (SRA) under accession number PRJNA1169526. The data are publicly available at: https://www.ncbi.nlm.nih.gov/sra/PRJNA1169526.
