# Supplementary figures and images for: Therapeutic target genes and regulatory networks of gallic acid in cervical cancer
Source: Front Genet. 2025 Jan 20;15:1508869. doi: 10.3389/fgene.2024.1508869 (PMC11789760; doi:10.3389/fgene.2024.1508869)

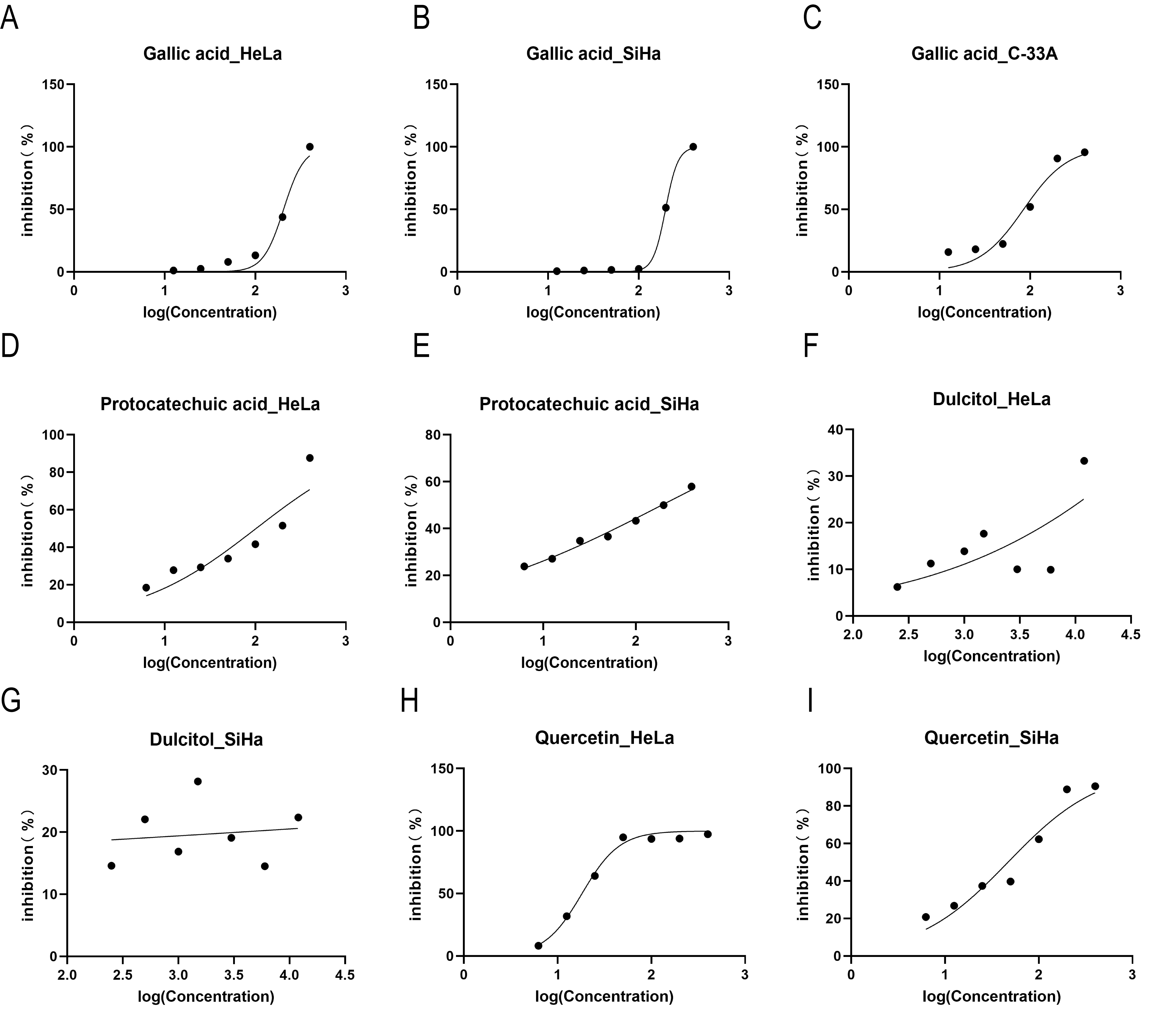

Supplement: Supplementary file 1 [file Image1.tif]
