# Supplementary material for: Therapeutic target genes and regulatory networks of gallic acid in cervical cancer
Source: Front Genet. 2025 Jan 20;15:1508869. doi: 10.3389/fgene.2024.1508869 (PMC11789760; doi:10.3389/fgene.2024.1508869)
Supplement: Supplementary file 2 [file DataSheet1.pdf]

Supplementary Material

1 Supplementary Data

To assess the inhibitory effects of gallic acid on cervical cancer cell lines, HeLa, SiHa, and C-33 A cells were treated with gallic acid at concentrations of 0, 12.5, 25, 50, 100, 200, and 400 μM (Figure 1A-C). For protocatechuic acid, HeLa and SiHa cells were treated with concentrations ranging from 0 to 400 μM (Figure 1D-E). Dulcitol treatments spanned concentrations from 0 to 12,000 μM on HeLa and SiHa cells (Figure 1F-G). Additionally, dulcitol was also tested on these cell lines with concentrations of 0, 6.25, 12.5, 25, 50, 100, 200, and 400 μM (Figure 1H-I). Based on the results, gallic acid and quercetin showed better inhibitory effects compared to other acid like protocatechuic acid.

Supplementary Figure 1. IC50 results of cervical cancer cells treated with different extract monomers

(A) IC50 assay treated HeLa cell line with gallic acid.

(B) IC50 assay treated SiHa cell line with gallic acid.

(C) IC50 assay treated C-33 A cell line with gallic acid.

(D) IC50 assay treated HeLa cell line with protocatechuic acid.

(E) IC50 assay treated SiHa cell line with protocatechuic acid.

(F) IC50 assay treated HeLa cell line with dulcitol.

(G) IC50 assay treated SiHa cell line with dulcitol.

(H) IC50 assay treated HeLa cell line with quercetin.

(I) IC50 assay treated SiHa cell line with quercetin.
